# Naturalist's Monthly Report

**Published:** 1811-11

**Authors:** 


### NATURALIST'S MONTHLY REPORT.

### SEPTEMBER.

Now golden fruits on loaded branches shine,
And grateful clusters swell with floods of wine;
Now blushing berries paint the yellow grove.

On the 1st and 2d of September the wind was northerly; from the 3d to the 19th inclusive, it was either easterly or north-west; on the 20th southerly, on the 21st south-west, on the 22d variable, on the 23d northerly, on the 24th north-west, from the 25th to the 27th westerly, on the 28th north, on the 29th westerly, and on the 30th north-west.

The weather from the 8th to the 15th was extremely hot, the sky being unobscured with clouds, and there having been no refreshing breezes, except for a few hours on the 11th. The only rain we had during the whole month fell on the 19th, 20th, 23d, 24th, 25th, 26th, 28th, and 30th.

The night of the 19th was stormy with thunder, and there was some thunder the next morning. There were strong gales on the 6th, 29th, and 30th; and squally weather on the 24th, 25th, and 26th.

September 1st. I have just been informed of a singular notion entertained, in some parts of this county, respecting toads, that, during the month of August, they are innoxious; and that, in consequence, the common people do not then so eagerly seek their destruction as at other times.

times. On the first of September, therefore, toads as well as partridges become again fair game.

Partridges this year are peculiarly scarce.

September 2d. Black grapes begin to change colour. Mulberries are in great profusion. The eclipse of the moon this evening was more beautiful than any eclipse that I recollect.

September 4th. About this time last year the swarms of wasps were innumerable, and these insects proved extremely injurious to the ripening fruits. This year there are very few indeed.

September 7th. Several of the autumnal plants are now in flower, particularly in the gardens, the Michaelmas daisies, and autumnal crocus; and of wild plants, the pale-flowered snakeweed (*polygonum pallidum*), orpine stonecrop (*sedum telephium*), common mugwort (*artemisia vulgaris*), and sea starwort (*aster tripolium*).

September 9th. Damsons are gathered. The second crops of clover are cut.

The bank martins (*hirundo rustica* of Linnæus) began to congregate amongst the reeds and sedge along the banks of the rivers; and particularly in the evenings, they are to be seen in immense numbers.

September 10th. In this part of Hampshire the barley harvest is completely ended.

September 13th. Gossamer floats. Winged ants come to life and fly abroad.

September 15th. The fishermen, for several evenings past, have been on the look out for herrings. The easterly winds, which have prevailed for several days past, are favourable for their arrival upon our shores; but hitherto, except a few stragglers, none have been caught.

September 18th. In consequence of the late dry and hot weather, the ponds and brooks begin to shrink. The water also in the rivers is very low.

September 20th. Martins and swallows congregate on the roofs. Thistle down floats. The goldfinches and other small birds eat these, and the seeds of numerous other weeds that are injurious to the farmers; thus rendering him much more service than he is aware of.

September 23d. This was a rainy day, and the farmers will now be able to begin their ploughing. The turnips also will be greatly benefitted; and vegetation altogether recovered from the effects of the late drought.

Hazel nuts and filberts are very scarce; and, with respect to walnuts, the trees, at least in this neighbourhood, are almost wholly destitute of them.

September 26th. Winter potatoes are taken up, and the crops upon the whole are very favourable. Grapes are gathered.

September 29th. This evening a considerable quantity of herrings was caught. On the following day they were sold for about seven pence per dozen.

Berberries are ripe. Wheat sowing is begun.

September 30th. The leaves of the walnut and lime trees begin to fall; and the heath and fern to turn brown.

The goat suckers have left us.

*Hampshire.*

